# What is a good doctor?

**DOI:** 10.1007/s10354-017-0597-8

**Published:** 2017-09-13

**Authors:** Verena Steiner-Hofbauer, Beate Schrank, Anita Holzinger

**Affiliations:** 1 Teaching Center, Medizinische Universität Wien, Spitalgasse 23, 1090 Vienna, Austria; 2 Karl Landsteiner Universität, Krems, Austria; 3 Medizinische Universität Wien, Vienna, Austria

**Keywords:** Good doctor, Medical education, Systematized review

## Abstract

Changes in medical curricula have led to a shift of focus in medical education. The goal was to implement a more practical approach to teaching and thereby create better doctors. However, the question of what makes a good doctor is not easy to answer. This article gives an overview on the literature about this topic. A systematized review and narrative synthesis were conducted including 20 articles about the features of good doctors. Qualitative and quantitative studies as well as questionnaires were included. These studies reported research involving students, doctors, patients, and nurses. The resulting characteristics of good doctors fell into six categories: (1) General interpersonal qualities, (2) Communication and patient involvement, (3) Medical competence, (4) Ethics, (5) Medical management, (6) Teaching, research, and continuous education. The different stakeholders showed different ideas of the concept of a good doctor. Interestingly, patients had a stronger focus on communication skills, whereas doctors put more emphasis on medical skills. Balancing this discrepancy will be a challenge for future medical education.

## Introduction

With the changes in medical curriculum, a new era of learning and teaching in medical education was started. Before these changes, specialist fields were taught separately from each other. In the new curriculum, the content should be horizontally and vertically integrated. Alongside with classical science, practical and communication skills have become significantly more important issues. These practical skills are taught in small groups in so-called “skills labs” or trained in simulated situations like in a simulated patient setting. Standardized learning target catalogues and practical exams—OSCEs—were developed to examine the new learning content in an appropriate way. The goal was to implement a more practical approach to teaching and thereby create better doctors, to prepare them specifically for the practical part of the job already during their education at the university—not afterwards in their first years of practice, as it was before.

During the process of creating learning target catalogues and appropriate ways of examination, it was determined which skills and knowledge the students should master at the end of their education to be able to work independently in a private practice or a hospital. But the question remains as to whether the new standards and ways of teaching lead to better doctors?

However, the question of what makes a good doctor is not easy to answer. It is not clearly defined what the essence of a good doctor is. The answer will most certainly vary depending on whom you ask because the different stakeholder groups have different needs and interests [1].

In a first approach to this topic, we wanted to know how the concept of a “good doctor” is covered in literature, which aspects and contents are connected to this concept and how different stakeholders like doctors, nurses and students rate or rank these aspects differently.

## Methods

This systematized review should give a summary and narrative analysis of the existing literature on the topic of “good doctor”. To broaden the scope, questionnaires and quantitative and qualitative data were included without any restrictions in quality or size of study population.

### Inclusion criteria

All quantitative and qualitative data were included if the explicit goal of the study was to find details on the concept of a “good doctor”. Primary studies in medical education and all medical disciplines were included without restrictions in quality or size of the study population. In qualitative studies all kinds of instruments for assessing attributes of good doctors were included, from standardized, validated questionnaires to simple questions. Included were all articles published in peer-reviewed or nonpeer-reviewed journals in English and German language and, in addition, open access questionnaires which measure patient satisfaction.

### Search methods for identification of studies

We searched six electronic databases from inception to 09 November 2015: Medline, EMBASE, PsychInfo, and Social Policy and Practice (accessed via Ovid1), CINAHL (accessed via EbscohostREF) and Web of Science. Databases were searched using the following terms, derived from scoping searches, and adapted to the requirements of the individual databases and interfaces: in keyword or title: (1) the terms good, great, fantastic, excellent, exceptional, competent, and professional adjacent within two positions with the terms doctor and physician, combining pairs with the term “OR”; (2) the terms good, great, fantastic, excellent, exceptional, competent, and professional directly adjacent to the terms anaesthetist, anesthetist, cardiologist, dermatologist, endocrinologist, gastroenterologist, gynaecologist, gynecologist, haematologist, haematologist, hepatologist, nephrologist, neurologist, obstetrician, oncologist, ophthalmologist, orthopaedist, orthopedist, paediatrician, paediatrician, psychiatrist, pulmonologist, radiologist, rheumatologist, surgeon, urologist, and virologist, combining all pairs with the term “OR”; (3) professionalism adjacent to the terms scale, assessment, inventory, questionnaire, evaluation, measure$ (measure “OR” measurement where truncation was not possible), instrument, rating, combining all pairs with “OR”; (4) competenc$ (competency “OR” competence where truncation was not possible) adjacent to the terms scale, assessment, inventory, questionnaire, evaluation, measure$ (measure “OR” measurement where truncation was not possible), instrument, rating, combining all pairs with “OR”; and in title only: (5) competenc$ (competency “OR” competence where truncation was not possible) combined with “AND” with the terms anaesthetist, anesthetist, cardiologist, dermatologist, endocrinologist, gastroenterologist, gynaecologist, gynecologist, haematologist, haematologist, hepatologist, nephrologist, neurologist, obstetrician, oncologist, ophthalmologist, orthopaedist, orthopedist, paediatrician, paediatrician, psychiatrist, pulmonologist, radiologist, rheumatologist, surgeon, urologist, and virologist, combined with “OR”, and (6) professionalism.

A broad range of search terms was chosen; at the same time the search strategy was restricted to words in title. The aim of this strategy was to narrow down this wide topic to target specifically relevant articles. A scoping search showed a broad range of literature on the topic of practical skills. This topic is explicitly not subject of this article.

### Data collection

**Fig. 1:**
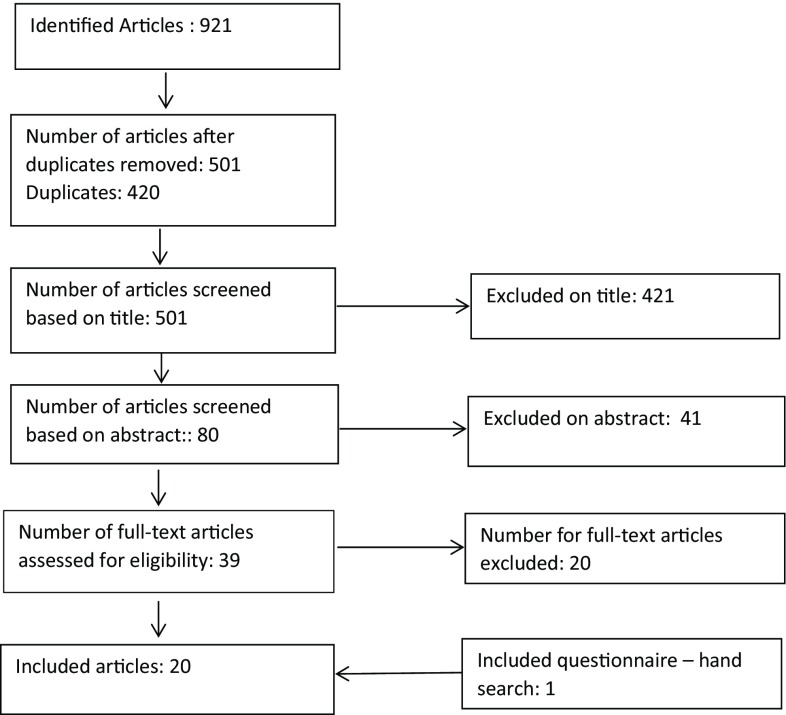
Flowchart of selection process

In a first step all duplicates were removed. Afterwards we excluded all articles with unsuitable titles including all articles in other languages than German or English. From the remaining articles the abstracts were reviewed. And in a last step all remaining papers were read and excluded or included based on the full content. Fig. 1 shows a flowchart of the selection process.

### Data extraction and analysis

All the data were extracted into an excel file. In qualitative studies the results—the attributes of good doctors—were extracted. In quantitative studies the content of the questions was used, but not the outcome of the studies. This is how we created a pool of equivalent statements. The different attributes found in the remaining articles were grouped together in six different categories. The authors agreed on the categories; disagreement was resolved by discussion and consensus.

## Results

Overall 20 articles were included in the analysis. Fig. 1 shows a flowchart of the selection process.

The included articles are scientific papers with quantitative and qualitative approach and/or free accessible questionnaires for the evaluation of patient satisfaction. The populations include the views of different stakeholder groups: doctors of different specialities (2 questionnaires, 6 studies), medical students (5 studies), patients (4 questionnaires, 4 studies), general population (5 studies), nurses (1 study), and others (6 studies). Three studies took place in UK. Studies conducted in Germany, Romania, Mozambique, Australia, Ireland, Singapore, Israel, Korea, the United States of America and Iran were included. The presented studies included more the 4000 people. Some of the articles included more than one stakeholder group. All studies are described in detail in Table 1.

**Table 1:** Included studies/questionnaires

Reference	Country	#	Sample	Method	Study outcome	Way of data collection
Oxford private medical practice [2]	UK	–	Patients	Open access questionnaire for patients	n.d.	Questionnaire
Fones CSL, Kua EH, Goh LG 1998 [3]	Singapore	274 400	General population / patients (Z*)	Quantitative, qualitative	Qualities and attributes of ideal doctors	Literature search, Delphi method, questionnaire
Klingenberg. 1999 [4]	International	–	Patients	Europep Patient Satisfaction Questionnaire	n.d.	Questionnaire
Cullen W, Bury G, Leahy M 2003 [5]	Ireland	599	General population (Z*)	Quantitative, qualitative	Attributes of good doctors	Interview
Mercer SW 2004 [6]	UK	–	Patients	Open access questionnaire for patients (CARE Consultation and Relational Empathy)	n.d.	Questionnaire
Schattner A, Rudin D, Jellin N [7]	Israel	445	Patients (Z+)	Quantitative	Good doctors, patients perspective	Questionnaire
Herzig S et al. 2006 [8]	Germany	83	Doctors	Qualitative	When is a doctor a good doctor?	Interview
Maudsley G, Williams EM, Taylor DCM 2007 [9]	UK	973	Students Future students	Quantitative, qualitative	Thoughts about the “good doctor”	Questionnaire
Gilles RA, Warren PR, Messias E, Salazar WH, Wagner PJ, Huff TA 2009 [10]	USA	189	Students	Qualitative	What makes a good doctor?	Focus groups
Lambe P, Bristow D 2010 [11]	UK	10 48	Doctors General Population (Z)	Quantitative	Attributes of a good doctor	Delphi-Method
Pfeiffer A, Noden BH, Walker ZA, Aarts R, Ferro J 2011 [12]	Mozambique	115 611	Students General population (Z)	Quantitative, qualitative	Beliefs of good and bad doctors	Interview, Questionnaire
Kliems H, Witt CM 2011 [13]	Germany	29 20 4	Observation Patients (Z) Doctors	Qualitative	Factors of the good doctor	Observation, Interview
General Medical Council 2012 [14]	UK	–	Patients	Open access questionnaire for patients	n.d.	Questionnaire
General Medical Council 2012 [14]	UK	–	Doctors	Open access questionnaire for patients (assessment of colleagues)	n.d.	Questionnaire
General Medical Council 2013 [14]	UK	–	Doctors	Open access questionnaire for doctors (self-assessment)	n.d.	Questionnaire
Iliescu L, Carauleanu A 2014 [15]	Rumania	160 80 80	Patients (Z) Students Nursing students	Quantitative, qualitative	Profile of a good doctor	Questionnaire
Miratashi Yazdi SN, Saharnaz N, Arbabi M, Majdzadeh R 2015 [16]	Iran	40 150	Patients (Z) Doctors	Quantitative, qualitative	Who is a good doctor?	Interview, Ranking
Cuesta-Briand B, Auret K, Johnson P, Playford D [17]	Australia	49	Students	Qualitative	Professionalism and “the good doctor”	Focus groups
Kim JH, Tor PC, King J, Seo JS 2015 [18]	Korea	140 184	Doctors Patients (Z)	Quantitative	What is a good psychiatrist?	Questionnaire
Bardgett RJM, Darling JC, Webster E, Kime N 2016 [19]	UK	28	Children (Z*)	Qualitative	What makes a good children’s doctor?	Interview

*n.d.* study outcome not defined, *Z* convenience samples from general population recruited in a hospital setting, Z* convenience samples from general population recruited in a nonclinical setting*, Z+ *random samples

### Population

In most of the studies convenience samples were used. That means that people selected themselves by voluntary participation. The populations marked with “Z” are convenience samples of patients. These people were approached in a hospital setting (in waiting areas or wards). The populations marked with “Z*” are convenience samples of general population; these people were recruited in a nonclinical setting (shopping mall, on the street), therefore they are considered as “general population” even if they may understand themselves as patients too since there is a good chance that most people have made “patient experience” of any kind during their past. The populations marked with “Z+” are random samples.

### Attributes of good doctors

We were able to summarize the attributes described in the included articles in six different superordinate categories, whose boundaries remain fuzzy: (1) General interpersonal qualities, (2) Communication and patient involvement, (3) Medical competence, (4) Ethics, (5) Medical management, (6) Teaching, research, and continuous education.

Most frequently, attributes of the categories “General interpersonal qualities” (*n* = 66) and “Communication and patient involvement” (*n* = 59) were mentioned, followed by “Medical competence” (*n* = 50), and “Ethics” (*n* = 48). “Medical management” (*n* = 32) and “Teaching, research, and continuous education” (*n* = 28) were least often mentioned. All counts are shown in Table 2.

**Table 2:** Categories and frequency of denomination

Attribute	*n*
General interpersonal qualities	66
Communication and patient involvement	59
Medical competence	50
Ethics	48
Medical management	32
Teaching, research, and continuous education	28

Most of the included articles and questionnaires include more than one category but all 20 included studies or questionnaires contained at least one item or question of the category “communication and patient involvement”. In all, 19 of the studies/questionnaires named a question or item of the category “General interpersonal qualities” and “Medical competence”, while 18 included “Ethics”. Questions or items regarding “Medical management” or “Teaching, research, and continuous education” were found only in half of the studies/questionnaires. The distribution of items/questions is shown in Table 3.

**Table 3:** Occurrence of categories per reference

		1	2	3	4	5	6
		General human qualities	Communication and patient involvement	Medical skills and competence	Ethics	Medical management	Teaching, research, and continuous education
	Reference						
1	General Medical Council (Self) [14]	X	X	X	X	X	X
1	General Medical Council (Patient)	X	X	X	X	–	–
1	General Medical Council (Coll)	–	X	X	X	X	X
1	Oxford private medical practice	X	X	X	X	–	–
1998	Fones CSL, Kua EH, Goh LG [3]	X	X	X	X	X	X
1999	Klingenberg A, Bahrs O, Szecsenyi J [4]	X	X	X	X	–	–
2002	Leahy W, Cullen W, Bury W [5]	X	X	X	X	–	–
2004	Mercer SW [6]	X	X	–	X	–	–
2004	Schattner A, Rudin D, Jellin N [7]	X	X	X	X	–	X
2006	Herzig S [8]	X	X	X	X	–	X
2007	Maudsley G, Williams EM, Taylor DCM [9]	X	X	X	X	X	X
2009	Gilles RA, Warren PR, Messias E, Salazar WH, Wagner PJ, Huff TA [10]	X	X	X	X	X	–
2010	Lambe P, Bristow D [11]	X	X	X	X	X	X
2011	Pfeiffer A, Noden BH, Walker ZA, Aarts R, Ferro J [12]	X	X	X	X	X	–
2011	Kliems. H, Witt CM [13]	X	X	X	–	–	–
2014	Iliescu, L, Carauleanu, A [15]	X	X	X	X	X	X
2014	Miratashi Yazdi SN, Saharnaz N, Arbabi M, Majdzadeh R [16]	X	X	X	X	X	–
2014	Cuesta-Briand B, Auret K, Johnson P, Playford D [17]	X	X	X	X	X	X
2015	Kim JH, Tor PC, King J, Seo JS [18]	X	X	X	X	X	X
2016	Bardgett RJM, Darling JC, Webster E, Kime N [19]	X	X	X	–	–	–

## Categories

### General interpersonal qualities

Being a practicing doctor is a profession that requires intensive interpersonal contact. For patients the contact with doctors is often a negative experience since it is often connected to pain, illness and uncertainty. This could be one of the reasons why people have high expectations in the interpersonal skills of their doctors.

The category “general interpersonal qualities” contains personal attributes and behaviour, which are not connected to medicine directly. This includes being friendly and nice, polite and cheerful. Making patients feel at ease, showing empathy and being able remain calm under pressure. This category also contains having a positive outlook on life, a good sense of humour, a well-balanced temper and love for people. These are all attributes we would wish to find in all people surrounding us, but especially in doctors.

### Communication and patient involvement

In an area where people with different backgrounds should decide together in often burdensome and stressful situations, communication is a task of enormous importance. Doctors have to listen carefully to the needs of their patients and share information in an appropriate way, tailored to the abilities of their patients, to reach satisfying outcomes.

The main topics in this category are attentive listening and clear and understandable explaining. This includes explaining tests and test results as well as answering questions honestly, open and in a language the patient can understand. Common rules of polite conversation, like giving enough time to speak about patient history and symptoms without interruptions belong to this category. The involvement of patients in medical decisions is only possible and useful if all necessary information is presented in an understandable way.

### Medical competence

This is the core competence of practicing doctors and the most extensive part of medical education is dedicated to the area of medical competences. Medical expertise, manual medical skills in treatment of patients, healing of illnesses and soothing discomfort and pain are included in this category. Examples include the following: taking a good history, find the suitable diagnosis and therapy, accuracy, experience as well as a holistic view on medical problems.

### Ethics

The doctor–patient relationship needs trust and honesty. This could be one of the reasons why people have high expectations about ethics and moral of their doctors. This category contains humbleness, honesty, integrity, trustworthiness and confidentiality as well as motivation and passion for work beyond obligation or financial interests. The ability to be self-reflective and the recognition of one’s own limitations are also part of this concept. Finally ethics include respect for different ethnicities and consideration of different religious and cultural believes.

### Medical management

Doctors, mainly in hospitals but in private practice too, have a list of duties beyond taking care of patients. Doctors take responsibility in leading teams, they represent the hospital and to some extent they represent the whole health system. To fulfill all these responsibilities, they need a set of characteristics that includes intelligence, flexible and independent thinking, decisiveness, good organisational skills and leadership qualities. They should be able to work well in a team and show respect for their colleagues, for students and (medical) staff members. A pleasant appearance and good personal hygiene are part of this category too.

### Teaching, research, and continuous education

Medicine as a field of science, where research and teaching are part of every day’s life, was mentioned least often in the included articles/questionnaires. Yet this is—without any question—an important part of medical profession. This category included research, teaching and supervision of students and colleagues as well as approachability for young colleagues and a commitment to a lifelong learning process.

### Different stakeholders, different interests

As far as we could derive from all included articles/questionnaires, there are different views on the importance of the attributes of good doctors in the different stakeholder groups.

### Patients

We included 48 items out of patient questionnaires; 24 of these items were matched to the category “communication and patient involvement”, “general interpersonal qualities” was mentioned 8 times as was “medical competence” (n = 8). Items concerning ethical aspects were named 7 times and only once was “medical management” mentioned. Communicative competences are therefore clearly in focus of patient satisfaction questionnaires.

### Students

Studies which targeted students’ opinions showed that in only one of the included 4 studies “competence in teaching” (being a good teacher) was rated under the top 5 attributes of a good doctor. The focus of medical students is on “medical competence” and “general interpersonal qualities”. Even for the students, who are directly affected by teaching skills, “medical competence” is rated higher.

### Doctors

Questionnaires and studies with focus on doctors’ opinions rated “medical competence” and “ethics” most important. “General interpersonal qualities” and “communication and patient involvement” are much less valued. In none of the included studies was communication rated as the most important attribute of a good doctor.

### Nurses

Only one article had nurses as a target population. There is not enough data for useful interpretation.

## Discussion

### Classification in categories

The classification of all attributes in the six categories: (1) General interpersonal qualities, (2) Communication and patient involvement, (3) Medical competence, (4) Ethics, (5) Medical management, (6) Teaching, research, and continuous education appeared conclusive and practical useful. It would also be possible to find other clusters or different labels for similar categories. For several items it was difficult to find only one suitable category. For example, patience in a conversation could be categorised as a communicative quality, but in context of physical examination it could be as a medical skill. Without any further information, patience is a virtue, desirable for all people and therefore it was categorised as a general interpersonal quality. The categories stated here are a useful tool to summarize the gathered information and build a useful framework. In this sense it was possible to find six basic attributes or cluster of attributes in the content of all used studies and questionnaires. For future research on the concept of “good doctor”, it could be useful to consider all 6 areas of interest. On this basis a questionnaire could be developed investigating the concept of “a good doctor” in the Austrian population. In adapted forms it could also be used in other stakeholder groups such as medical students, nurses and other medical professionals to gain a holistic view on this concept.

The different stakeholders showed different preferences in categories. This could be a reason for dissatisfaction and misunderstandings between patients, doctors and students in everyday hospital work. The category “communication and patient involvement” shows this very clearly.

In all the questionnaires used to measure patient satisfaction, communication was a main topic. Whenever people had the chance to rate attributes at their own discretion, it was rated in almost all cases under the top 5 attributes too [5, 7, 13, 18]. General interpersonal qualities are also highly demanded [5, 7, 12, 13, 18, 19].

For medical education this could be a strong sign towards communication training because communication seems to be a key aspect of patient satisfaction. How medical procedures, therapies and test results are explained, how long a patient has time to talk about his or her medical issues, or how a doctor reacts to questions, is part of good communication. It should not be left to chance if and how this core element of doctor–patient relationship works out. Fallowfield et al. [20] underline the importance of communication quality in their study on oncologists and their patients. They show that due to a three-day communication training they could improve the quality of questions significantly, reduce the rate of leading questions, and raise empathic behaviour and appropriate reactions.

A very important aspect is communication between doctors and nurses (and other medical professionals). If there are fewer misunderstandings between those two groups, patient care is more effective [21]. The best way to reach this goal of respectful teamwork and fluent communication is to introduce interprofessional education programmes. Until then, Curtis et al. [21] suggest special training for structured doctor–nurse communication.

Since 2001, trainings for shared decision making are promoted by the German state. Doctors should learn to ask patients for their tendencies towards participating in medical decisions and how patients can be included in decision making regarding to their abilities and needs [22].

Many universities have communication training implemented in their curriculum. These lessons are often conducted in a role play setting with simulated patients. Simulated patients or standardized patients are professional or lay actors who exercise different conversations on various topics together with the students.^1^ It is also possible to include general interpersonal qualities in a curriculum. An adequately friendly welcome, keeping eye contact, or showing empathy through addressing perceived emotions are skills which can be taught and learned during trainings or in practical education.

In the curriculum of the Medical University of Vienna the mandatory element “soziale Kompetenz” (social competence) is implemented. Intended learning outcomes of this training are adequate communication, empathy, appreciation and professional behaviour in an interdisciplinary team. The students have the chance to learn those social competences in contact with people in need of care in a nursing home.

A very important aspect remained unaddressed: Asking patients about their own beliefs and concepts of sickness, health and the origin of their disease as well as asking about complementary medicine and “self-treatment”. This whole area was not covered in the included literature, despite there being strong evidence that this can be an important part of recovery [23, 24].

Doctors themselves emphasise medical competence as their most important attribute. This is without question an area of great impact and a core competence. Patients do not rank this category as high as doctors—maybe because patients as lay persons do not have the skills to evaluate the quality of their doctor’s work in the sense of medical competence and therefore just assume that every doctor is qualified and reliable.

Nurses and other medical professionals could contribute important insights in the everyday work of doctors and the day-to-day experience of patients. This could be of greatest interest for the definition of what makes a good doctor.

To assess the real differences between all stakeholder groups, it is necessary to collect data on representative samples with standardised and similar methods. This study can provide the theoretical groundwork.

## Footnotes

1 The simulated patient programmes of the Charité in Berlin (https://lernzentrum.charite.de/ressourcen/simulationspatienten/) and the Medical University of Vienna (https://teachingcenter.meduniwien.ac.at/abteilungen/methodik-und-entwicklung/schauspielpatientinnen-programm/) serve as examples for many other similar programmes.
